# KCNQ1OT1: An Oncogenic Long Noncoding RNA

**DOI:** 10.3390/biom11111602

**Published:** 2021-10-29

**Authors:** Patrice Cagle, Qi Qi, Suryakant Niture, Deepak Kumar

**Affiliations:** Julius L. Chambers Biomedical Biotechnology Research Institute, North Carolina Central University, Durham, NC 27707, USA; pcagle@nccu.edu (P.C.); qqi@nccu.edu (Q.Q.); sniture@nccu.edu (S.N.)

**Keywords:** human cancers, competing endogenous RNA, long noncoding RNA, KCNQ1OT1

## Abstract

Long noncoding RNAs (lncRNAs) are transcripts greater than 200 nucleotides that do not code for proteins but regulate gene expression. Recent studies indicate that lncRNAs are involved in the modulation of biological functions in human disease. KCNQ1 Opposite Strand/Antisense Transcript 1 (KCNQ1OT1) encodes a lncRNA from the opposite strand of KCNQ1 in the CDKN1C/KCNQ1OT1 cluster that is reported to play a vital role in the development and progression of cancer. KCNQ1OT1 regulates cancer cell proliferation, cell cycle, migration and invasion, metastasis, glucose metabolism, and immune evasion. The aberrant expression of KCNQ1OT1 in cancer patients is associated with poor prognosis and decreased survival. This review summarizes recent literature related to the biological functions and molecular mechanisms of KCNQ1OT1 in various human cancers, including colorectal, bladder, breast, oral, melanoma, osteosarcoma, lung, glioma, ovarian, liver, acute myeloid leukemia, prostate, and gastric. We also discuss the role of KCNQ1OT1 as a promising diagnostic biomarker and a novel therapeutic target in human cancers.

## 1. Introduction

Cancer remains a leading cause of death and a significant public health problem worldwide, with approximately 19.3 million new cancer cases and 10 million deaths occurring in 2020 [1]. In the United States alone, 1.8 million new cancer cases and over 600,000 cancer deaths are expected in 2021 [2]. Cancer is a multifactorial disease associated with genetic mutations, chromosomal aberrations, and epigenetic changes [3]. In recent decades, significant efforts have been invested in understanding biological molecules involved in the development and progression of various human cancers. 

Long noncoding RNAs (lncRNAs) are a large and diverse class of transcribed RNA molecules with a length of more than 200 nucleotides that do not code for a protein product. [4]. It has been estimated that the human genome contains approximately 56,946 lncRNA genes [5], about 2700 microRNAs (miRNAs)-coding genes [6], and over 20,000 protein-coding genes [7]. LncRNAs are transcribed by RNA polymerase type II (RNAP2) and are controlled by the transcriptional activators of the SWI/SNF complex. They undergo post-transcriptional modifications such as splicing, 3′ polyadenylation, and 5′ 7-methylguanosine capping [8,9]. Although lncRNA expression levels are lower than messenger RNA (mRNA) and their sequences are poorly conserved, lncRNAs are functional molecules [10]. LncRNAs may behave like tumor suppressors or oncogenes that regulate gene expression at different levels through their interaction with DNA, miRNA, mRNA, and proteins [11,12]. 

LncRNAs can control protein-coding and noncoding gene expression and function in tissue and developmental-stage specific manner by several mechanisms, including regulation of chromatin remodeling, RNA splicing events, transcriptional activation/repression, mRNA stability and translation by homologous base pairing, and post-transcriptional regulation of protein activity [11,13,14,15,16]. LncRNAs can interact with a variety of partners such as RNA/DNA-binding proteins, transcription factors, chromatin-modifying complexes, RNA transcripts, mature mRNA, microRNA (miRNA), DNA, and chromatin [17]. Moreover, lncRNAs harboring microRNA recognition elements also function as competing endogenous RNAs (ceRNAs) that bind to and “sponge” miRNAs, downregulating and preventing them from binding to and regulating their target mRNAs [12,18,19]. This interaction between the two classes of RNA is mediated by the RNA-induced silencing complex (RISC) and determines the level of post-transcriptional regulation of gene expression [20]. LncRNAs have been shown to modulate multiple cancer hallmarks, including cell cycle regulation, cell proliferation, angiogenesis, migration, invasion, epithelial-to-mesenchymal transition (EMT), metastasis, apoptosis, and cancer stem cell differentiation and cancer-associated immune responses [8,10,14,21]. In this review, we focus on LncRNA KCNQ1OT1 and its molecular and biological functions in human cancer. 

## 2. Basic Characteristics of Human Chromosome 11p15.5 and lncRNA KCNQ1OT1 Gene

**Figure 1:**
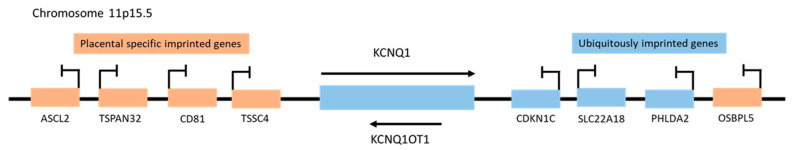
Schematic representation of imprinted gene clusters on human chromosome 11p15.5.

KCNQ1 Opposite Strand/Antisense Transcript 1 (KCNQ1OT1), also known as KCNQ1 overlapping transcript 1, or LIT1, is a 91 kb un-spliced lncRNA located on chromosome 11p15.5 (Figure 1). The KCNQ1OT1 gene is part of a cluster of genes that undergo genomic imprinting, an epigenetic modification involving parent-specific gene expression modification. Genomic imprinting plays a critical role in fetal growth and development and is regulated by a nearby region of DNA known as imprinting center 2 (IC2) or KvDMR, which undergoes differential methylation [22,23]. The human CDKN1C/KCNQ1OT1 cluster exists as imprinted genes, expressing only one copy, with the allele activity depending on the parental origin. The paternally expressed KCNQ1OT1 transcript originates from intron 11 and is antisense to its associated protein-coding gene, Potassium Voltage-Gated Channel Subfamily Q Member 1 (KCNQ1) [3,24,25,26]. The antisense lncRNA KCNQ1OT1 promoter maps to KCNQ1 imprinting control regions, methylated on the maternal chromosome but un-methylated on the paternal chromosome.

Genes located near the KCNQ1OT1 promoter (KCNQ1, CDKN1C, SLC22A18, and PHLDA2) that are imprinted both in the embryo and extra-embryonic tissues such as the placenta are ubiquitously imprinted genes, whereas the placental specific imprinted genes ASCL2, TSPAN32, CD81, TSSC4, and OSBPL5, and are only imprinted in the placenta (Figure 1) [16]. The ubiquitously expressed KCNQ1OT1 is more frequently localized in the nucleus, interacts with chromatin complexes, and regulates the genomic imprinting of multiple genes through bidirectional transcription-mediated silencing in cis [26,27,28]. Thus, the DNA sequences of the KCNQ1 and KCNQ1OT1 genes are “read” in opposite directions and have very different functions. LncRNA KCNQ1OT1 is expressed in every tissue [29] and regulates genes vital for normal growth and development before birth, as well as postnatal behavior [26,30]. However, deletion of its promoter or early transcript termination results in a loss of KCNQ1OT1 and a disruption of imprinting in the CDKN1C/KCNQ1OT1 domain, which can lead to growth-related disorders (ex. Beckwith-Wiedemann syndrome) and cancer, as well as bi-allelic expression of the entire KCNQ1 domain [31].

## 3. KCNQ1OT1 in Human Cancers

**Figure 2:**
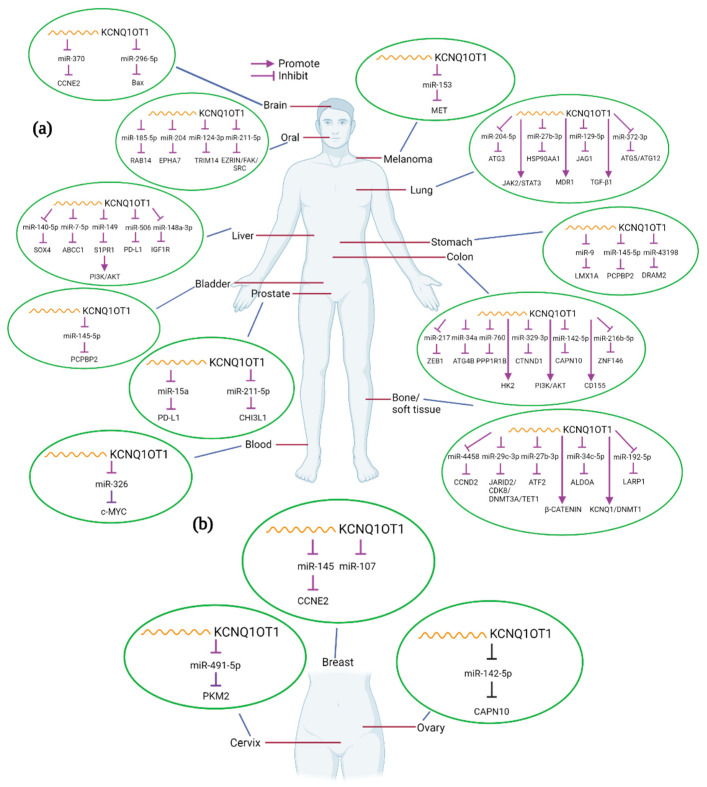
Interactions between KCNQ1OT1 and miRNA/genes in different kinds of human cancers. KCNQ1OT1 can interact with various miRNAs and genes in different tumor types (**a**), including female-specific cancers (**b**). Created with BioRender.com.

In the present review, we will explore current knowledge on the role of KCNQ1OT1 in the development of various human cancers. We will thoroughly discuss the molecular and mechanistic role of KCNQ1OT1 in modulating oncogenic and biological functions, regulating cancer cell signaling mechanisms, and describe how its expression correlates to clinical features (Table 1). The interaction of KCNQ1OT1 and miRNAs or/and proteins and the potential targets in different cancers are summarized in Figure 2.

### 3.1. Colorectal Cancer

Colorectal cancer (CRC) is one of the most common cancer types and a leading cause of cancer deaths worldwide for both sexes combined. In the United States (U.S.), the overall 5-year survival rate for localized stage CRC is 90%. In comparison, the survival rate for patients diagnosed with regional and distant-stage disease drops to 71% and 14%, respectively [32]. Therefore, new molecular markers for early diagnosis of CRC are still needed to reduce the incidence and mortality of CRC. KCNQ1OT1 is upregulated in colorectal (CRC) cancer tissues and cells, and its overexpression is associated with disease progression and poor prognosis in patients with CRC [33,34,35]. Furthermore, silencing of KCNQ1OT1 decreased the oncogenic properties of CRC DLD1 and SW480 cell lines [33]. Interestingly, silencing of KCNQ1OT1 also decreased Bcl-2, MMP9, Cyclin D1, while the levels of cleaved Caspase-3 proteins and E-cadherin proteins were increased in SW480 and LS1034 CRC cells [36]. 

KCNQ1OT1 mediated drug resistance and was found to be overexpressed in tissue obtained from CRC patients that showed resistance to the drug methotrexate (MTX) [37]. KCNQ1OT1 was also found to be overexpressed in various MTX-resistant CRC cell lines, demonstrating its role in decreased therapy effectiveness and chemoresistance [37]. Knockdown of KCNQ1OT1 reduced the proliferation of MTX-resistant HT29 and Caco2 CRC cells and induced cell cycle arrest and apoptosis by regulating the miR-760/protein phosphatase 1 regulatory inhibitor subunit 1B axis [35]. Mini et al. proposed that KCNQ1OT1 may be used as a molecular biomarker to identify patients with stage III CRC who may significantly benefit from adjuvant fluorouracil-based chemotherapy [35]. The possibility of identifying a biomarker predictive of response to drugs and preventing unnecessary treatment to unresponsive CRC patients would be a tremendous advancement in CRC therapy. High KCNQ1OT1 positively correlates with CD155 expression and is associated with poor prognosis of CRC patients, mediating CD8+ T cell exhaustion in CRC. In addition, KCNQ1OT1 has been reported to play a role in metabolic reprogramming in CRC. Chen et al. demonstrated that KCNQ1OT1 modulates HCT116 and SW48 CRC cell proliferation in glucose metabolism reprogramming by stabilizing hexokinase 2 (HK2) [38]. Together, studies suggest that higher levels of KCNQ1OT1 in CRC patients could reduce the effectiveness of some chemotherapeutic regimens and may be useful as a predictive biomarker of drug response, a prognostic indicator, and an immune therapy target. 

### 3.2. Glioma

Glioma is one of the most common and life-threatening adult brain tumors, with a median survival time from 12.1 to 14.6 months [39]. Gain- or loss-of-function studies have revealed that lncRNAs may play critical roles in regulating glioma development and progression by acting as tumor suppressors or oncogenes [40]. Gong et al., found that KCNQ1OT1 was upregulated in glioma patient tissues compared with normal brain tissues and was positively correlated with the histopathological grades of gliomas [41]. Knockdown of KCNQ1OT1 inhibited cell proliferation, migration and invasion, and promoted apoptosis in U87 and U251 glioma cells. In addition, KCNQ1OT1 promoted the aggressiveness of glioma cells through the miR-370/CCNE2 (cyclin E2) signaling pathway [41] (Table 1). Furthermore, knockdown of KCNQ1OT1 combined with overexpression of miR-370 reduced tumor growth [41]. The results of this study suggest KCNQ1OT1 might contribute to glioma malignancy, and targeting the KCNQ1OT1/miR370/CCNE2 axis may be a promising treatment option for glioma patients.

### 3.3. Oral Cancer

#### 3.3.1. Oral Squamous Cell Carcinoma 

There are more than 53,000 new cases of head and neck cancers diagnosed each year and nearly 11,000 cancer-related deaths in the U.S. [47]. Squamous cell carcinoma (SCC) accounts for the majority of cases (~90%), often occurring within the oral cavity and oropharynx. Oral squamous cell carcinoma (OSCC) is the most common oral malignancy, with nearly 378,000 new cases reported annually worldwide [48]. Patients with OSCC typically experience delayed detection and a poor prognosis. Despite progress in research and therapy, survival has not improved significantly in the last few decades.

Furthermore, the available treatments are often expensive and result in disfiguration and lower quality of life to the patient. The lack of confirmed biological markers, including lncRNAs, continues to present a challenge in the early diagnosis of OSCC and is needed to reduce the burden of OSCC. A clinical study reported that KCNQ1OT1 expression is upregulated in OSCC tissues compared to adjacent normal tissues and cell lines and is correlated with poor survival of OSCC patients [14]. Knockdown of KCNQ1OT1 suppressed cell migration and invasion and promoted apoptosis in SCC15 and HSC-3 cells by modulating miR-185-5p and its downstream target Rab14 (RAB14, a Ras oncogene family member) [14]. Though this study had a limited study population, results suggest that it may play a role in the malignant behavior of OSCC cells [14] (Table 1). Animal models may also be helpful to further explore the biological function of KCNQ1OT1 in OSCC in vivo. 

#### 3.3.2. Tongue Squamous Cell Carcinoma 

Tongue cancer is a common malignant tumor in the oral and maxillofacial region, most of which is SCC. Tongue SCC (TSCC) is an aggressive disease with a favorable prognosis in the early stages but not in later stages, demonstrating the need for better prognostic tools. Cisplatin is one of the chemotherapy drugs utilized in patients with TSCC. Although nearly 80% of patients respond positively to cisplatin and overall survival rates are improved, 70% become resistant to cisplatin after initial treatment [16]. KCNQ1OT1 was found to be upregulated in chemo-resistant TSCC tissues and cisplatin-resistant TSCC cells. In addition, KCNQ1OT1 can upregulate Ezrin, a protein differentially expressed in aggressive cancer types and associated with poor survival [49], and promote cisplatin resistance of CAL27-res and SCC9-res cells through binding with miR-211-5p (Table 1). Knockdown of KCNQ1OT1 also reduced CAL27 and SCC9 cisplatin-resistant cell proliferation as well as tumor growth and weight in vivo via the miR-124-3p/TRIM14 axis [16]. Though molecular biomarkers are not currently clinically approved for TSCC, KCNQ1OT1 could add value as a prognostic factor or therapeutic target for TSCC.

#### 3.3.3. Maxillary Sinus Squamous Cell Carcinoma 

Maxillary sinus squamous cell carcinoma (MSSCC) is a rare malignancy located near the oral cavity and accounts for 3% of all head and neck cancers and 80% of all paranasal sinus cancers [50]. Santos et al. reported that the median survival time for patients with MSCC was 14 months, and the overall 1- and 5-year survival rate was 57.9% and 17.7%, respectively. Therefore, it is vital to understand the molecular mechanisms and identify new biomarkers involved in MSSCC pathogenesis. MiR-204 suppressed the expression of the Eph receptor A7 (EphA7) protein and decreased IMC-3 MSSCC cell proliferation, migration, and invasion (Table 1). Therefore, the KCNQ1OT1/miR-204/EphA7 axis may contain potential therapeutic targets for MSSCC. However, more comprehensive studies utilizing a varied population of MSSCC patients are needed to better understand the correlation of KCNQ1OT1 expression with tumor size, differentiation, or prognosis in this patient population. 

### 3.4. Acute Myeloid Leukemia 

Acute myeloid leukemia (AML) is a heterogeneous cancer of the blood characterized by uncontrolled proliferation and poor cell differentiation of myeloid progenitor cells and infiltration of malignant cells into the bone marrow, peripheral blood, or other tissues [51,52]. AML is the most common form of acute leukemia among adults, mainly affecting the older population (age >65) [53]. With the rising morbidity and mortality in older patients, it is necessary to explore new biomarkers for diagnosis, prognosis, and therapeutic targets of AML to improve patient outcomes. A recent study has shown that dysregulation of KCNQ1OT1 is associated with poor survival rates in patients with AML [54]. Su et al. found that KCNQ1OT1 was upregulated in Adriamycin (ADR)-resistant AML tissue and cells compared to ADR-sensitive tissue and cells. Also, knockdown of KCNQ1OT1 reduced HL60 and K562 ADR resistance, inhibited cell proliferation, migration, and invasion, but promoted apoptosis of ADR-resistant AML cells via the miR-193a-3p/Tspan3 axis [55] (Table 1).

### 3.5. Osteosarcoma

Osteosarcoma (OS) is the most common form of bone cancer, affecting children, adolescents, and young adults. Approximately 85% of patients with OS present with metastatic disease, stressing the urgent need to delineate the molecular mechanisms involved in OS progression and identify more effective treatment options. LncRNAs have been previously reported to be associated with the progression of OS. KCNQ1OT1 expression is increased in osteosarcoma patient tissue and is associated with OS progression and decreased overall survival. A recent study by Qi et al. reported that knockdown of KCNQ1OT1 decreased cell proliferation, migration, and invasion while promoting apoptosis and chemo-sensitivity of OS MG-63 cisplatin-resistant cells to cisplatin through the upregulation of KCNQ1 via DNA methyltransferase 1 (DNMT1) [20] (Table 1). Downregulation of KCNQ1OT1 inhibits OS cell proliferation, invasion, and resistance to fluorouracil (5-FU) by regulating miR-129-5p-mediated La-related protein 1 (LARP1). Multidrug resistance is one of the biggest obstacles in the treatment of OS [56], and KCNQ1OT1 may be a drug resistance-related lncRNA and a promising target to circumvent chemoresistance. 

### 3.6. Breast Cancer

Breast cancer (BRCA), a heterogeneous and complex disease, is one of the most prevalent cancer types globally [47,57]. BRCA disproportionally presents with a more aggressive phenotype in the African American population. LncRNAs have shown promise as potential prognostic biomarkers because of their role as functional regulators in BRCA tumorigenesis [26]. Reports suggest that KCNQ1OT1 is overexpressed in BRCA tissues and cellular models. Recent studies by Zhang et al. using chromogenic in situ hybridization show that KCNQ1OT1 is upregulated in invasive BRCA (IBRCA) and ductal carcinoma in situ (DCIS), compared to normal adjacent breast tissues. Interestingly, KCNQ1OT1 was not upregulated when comparing IBRCA and DCIS, suggesting that upregulation is an early event in BRCA progression. KCNQ1OT1 also promotes tumor growth in vitro and in vivo [25]. The study further pointed out that KCNQ1OT1 targeted miR-145/CCNE2, promoted cell cycle progression, increased migration and invasion, and inhibited cell apoptosis in MCF7 and SKBR-3 BRCA cells [25]. 

### 3.7. Liver Cancer

#### 3.7.1. Hepatocellular Carcinoma

Liver cancer is the second leading cause of cancer-related deaths worldwide, with more than 905,000 new cases reported every year [48]. Liver cancer involves several types, including hepatocellular carcinoma (HCC) and cholangiocarcinoma (CCA), commonly known as bile duct cancer, which develops in the small, tube-like bile ducts in the liver. Although early-stage HCC can be effectively treated by liver transplantation or curative surgery, it is not feasible for 60–80% of HCC patients [29]. Treatment for advanced stages of HCC also presents challenges due to disease relapse after therapy and drug resistance [58]. KCNQ1OT1 is upregulated in OXA-resistant hepatocellular carcinoma (HCC) patient tissues, compared with OXA-sensitive tissues. In addition, it has been reported to modulate OXA resistance through the miR-75p/ABCC1 (ATP Binding Cassette Subfamily C Member 1) axis in HepG2 and Huh HCC cells, indicating that KCNQ1OT1 may be a novel therapeutic target for HCC [28]. Chen et al. found that KCNQ1OT1 drives SMMC-7721 and Huh7 HCC cell invasion and migration while knockdown of KCNQ1OT1 inhibits tumor growth by acting on sphingosine-1-phosphate receptor 1 (S1PR1) through miR-149 [29] (Table 1). Thus, elucidating the role of KCNQ1OT1 in HCC could provide new therapeutic alternatives and improvement in prognoses. 

#### 3.7.2. Cholangiocarcinoma

The prognosis for patients with CCA is abysmal due to late diagnosis and tumor resistance to chemotherapy and radiotherapy, emphasizing the need for new therapeutic targets and novel biomarkers [59]. Sun et al., reported that KCNQ1OT1 was highly expressed in CCA tissues and cells, and its overexpression was correlated with poor survival in CCA patients [27]. The miR-140-5p-SOX4 (SRY-Box Transcription Factor 4) signaling pathway was shown to correlate with KCNQ1OT1 and suppress CCLP1 and RBE cell growth and invasion (Table 1). There is a lack of appropriate biomarkers for early detection and asymptomatic clinical presentation in the early stages of CCA. Therefore, the prognosis of patients with CCA is extremely poor due to diagnosis at advanced stages and the high recurrence rate [59,60,61]. As the survival rate for CCA is between 5–10%, further studies are necessary to determine if KCNQ1OT1 may be an important new biomarker and therapeutic target in reducing mortality in patients with CCA [59,60]. The effect of KCNQ1OT1 on other oncogenes associated with CCA, such as KRAS, FGFR2, IDH1, and IDH2, should also be investigated [60]. 

### 3.8. Bladder Cancer

Bladder cancer (BC) affects nearly half a million men and women annually and causes nearly 213,000 deaths worldwide [48]. Although the implementation of new technologies has improved BC treatment, patients in advanced stage BC are still confronted by a challenging prognosis, with a survival rate of only 5% [32,47]. Therefore, it is imperative to find new biomarkers or therapeutic targets for BC. Similar to CRC, recent research has demonstrated that KCNQ1OT1 is upregulated in BC tissues and cells. Knockdown of KCNQ1OT1 inhibited T24 and HT-1197 BC cell proliferation, colony formation ability, migration, and promoted apoptosis [32]. 

### 3.9. Lung Cancer

Lung cancer (LC) is now the second most common cancer in males and females worldwide after female breast cancer but remains the highest reported mortality [48]. LC is classified histologically into two main subtypes, small-cell LC (SCLC) and non-small-cell LC (NSCLC). Studies reveal that KCNQ1OT1 is overexpressed in NSCLC patients and is associated with a poor prognosis [35,62]. Progression-free and overall survival is significantly shorter in patients expressing high levels of KCNQ1OT1 than in patients expressing low levels of KCNQ1OT1, suggesting it may be a potential molecular marker for assessing prognosis in NSCLC [62]. ]

Kang et al. found that knockdown of KCNQ1OT1 promoted apoptosis suppressed proliferation and autophagy in NSCLC cells, and inhibited tumor growth in vivo. Furthermore, KCNQ1OT1 regulated NSCLC progression by binding to miR-204-5p and miRNA-27b-3p, modulating the expression of autophagy-related gene 3 (ATG3) and heat shock protein 90 alpha family class A member 1 (HSP90AA1), respectively [33,35]. 

Ren et al. showed that KCNQ1OT1 was significantly upregulated in paclitaxel-resistant lung adenocarcinoma (LAD) tissues and A549/PA cells compared with that in paclitaxel-sensitive lung tissues and cells. Knockdown of KCNQ1OT1 inhibited cell proliferation and invasion, promoted apoptosis, and decreased chemoresistance to paclitaxel by downregulating MDR1 in A549 LAD cells, suggesting that KCNQ1OT1 may be a potential therapeutic target for paclitaxel-based chemotherapy in LAD [34]. Interestingly, KCNQ1OT1 has also been shown to exert anti-tumor effects. Its overexpression in early-stage NSCLC inhibited cell proliferation and tumor growth and is associated with a favorable prognosis (Table 1). This finding suggests that KCNQ1OT1 may have anti-tumor effects in patients in the early stages of LC [40]. Together, these findings highlight the potential stage-specific prognostic and diagnostic utility of KCNQ1OT1, but additional studies are needed to confirm these findings. 

### 3.10. Melanoma

Melanoma, the most lethal form of skin cancer, is an aggressive disease with high morbidity and mortality. The incidence of melanoma continues to increase, with over 287,000 people projected to have developed melanoma in 2018 worldwide [41,57]. Despite advances in drug discovery and targeted molecular therapy, the prognosis is still unfavorable for those with distant metastasis, with a 5-year survival rate of only 25% [47]. LncRNAs have been linked to the development and progression of melanoma [63,64,65]. Guo et al. showed that KCNQ1OT1 was aberrantly upregulated in melanoma patient tissues compared with adjacent normal tissues, and patients with high levels of KCNQ1OT1 had a worse overall survival than those with low levels of KCNQ1OT1 [41]. 

### 3.11. Ovarian Cancer

In the U.S., ovarian cancer (OC) is a frequently occurring cancer type in the female reproductive system, with a poor survival rate (30%) due to nearly 75% of patients being diagnosed at late stages [47,66]. Therefore, new tumor markers are still needed for the early diagnosis of OC. The expression KCNQ1OT1 is elevated in A2780 OC cells, and Luo et al. reported that upregulation of KCNQ1OT1 promoted ovarian cell proliferation, migration, and cell cycle progression by activating β-catenin [67]. Furthermore, KCNQ1OT1 was found to target the miR-142-5p/CAPN1 (Calpain 1) axis to regulate cell proliferation, colony formation, and invasion in SKOV3 and OVCAR3 OC cells [9]. In addition, KCNQ1OT1 promoted OC progression and supported in vivo tumor formation via targeting the miR-212-3p/LCN2 (Lipocalin-2) axis [68] (Table 1). Overall, these results suggest that KCNQ1OT1 may play a role in developing OC and could be utilized as a biomarker to detect this cancer at earlier stages. 

### 3.12. Cervical Cancer

Cervical cancer is the fourth most common cancer among women and causes nearly 342,000 deaths worldwide [48]. Despite being one of the most preventable cancers through the HPV vaccine and screening, cervical cancer is the second leading cause of cancer death in women aged 20 to 39 years [69]. 

KCNQ1OT1 is upregulated in cervical cancer (CC) patient cancerous tissues and cell lines, and its high expression is significantly correlated with increased tumor volume and poor differentiation in cervical cancer patients. Upregulated KCNQ1OT1 also mediates the progression and radioresistance of CC by modulating the miR-491-5p/pyruvate kinase M1/2 (PKM2) axis [10]. KCNQ1OT1 knockdown inhibited cancer hallmarks, including cell proliferation, metastasis, and radioresistance.

### 3.13. Gastric Cancer

Gastric cancer (GC) is the sixth most common cancer worldwide and the third leading cause of cancer-related mortality, with nearly 1.1 million deaths in 2018 [57]. Although clinicians currently use several tumor markers for early detection of GC, the specificity and sensitivity of these biomarkers are not efficient. Numerous studies have shown that lncRNAs play significant roles in gastric cancer progression at the transcriptional and post-transcriptional levels, typically resulting in overexpression [70]. Zhong et al. revealed that the expression of KCNQ1OT1 was increased in GC patients and cell lines. The silencing of KCNQ1OT1 inhibited GC tumor growth, reduced cell proliferation, and induced GC cell apoptosis by binding to miR-145-5p, modulating the expression of ADP-Ribosylation Factor 6 (ARF6) [45] (Table 1). However, another study reported that KCNQ1OT1 is downregulated in human GC patient tissues compared to controls. Overexpression of KCNQ1OT1 in AGS primary GC cells inhibited cell survival, proliferation, migration, and invasion but induced apoptosis by downregulating miR-9 and upregulating LMX1A (LIM homeobox transcription factor 1, alpha) [44]. These studies suggest that the function of KCNQ1OT1 may vary in different types and stages of GC. Further studies are warranted to determine its effectiveness as a biomarker for GC prognosis and treatment.

## 4. Regulatory Mechanisms of KCNQ1OT1

### 4.1. Impact of KCNQ1OT1 on microRNA Regulation

LncRNAs acting as ceRNAs with miRNAs are the most frequently identified biological function of lncRNAs and has been associated with various cancers, such as lung [33,71], prostate [72], ovarian [9,73], colorectal [74], and glioblastoma multiforme [75]. By sponging miR-217, KCNQ1OT1 upregulated zinc finger E-box binding homeobox 1 (ZEB1) and regulated CRC cell proliferation, migration, and EMT formation [1] (Table 1). Similarly, the miR-329-3p/CTNND1 (Catenin delta-1) axis was demonstrated to interact with KCNQ1OT1 to modulate SW480 and LS1034 CRC cancer cell proliferation, migration, invasion, and apoptosis [4]. In a xenograft mouse model, knockdown of KCNQ1OT1 inhibited CRC cell growth and decreased tumor volume, while overexpression of KCNQ1OT1 induced protective autophagy and chemoresistance to oxaliplatin (OXA) by sponging miR-34a and upregulating autophagy-related 4B (Atg4B) [2]. Sun et al., showed that KCNQ1OT1 acted as a sponge of miR-204 in the progression of MSSCC and explored the role of ceRNA regulation of the KCNQ1OT1/miR-204/EphA7 axis [17]. Cell-based studies indicate that KCNQ1OT1 knockdown inhibited cell proliferation and promoted apoptosis and cell differentiation in HL-60 and U937 AML cells by acting as a ceRNA for miR-326 and targeting c-Myc (Myc proto-oncogene, basic helix-loop-helix (bHLH) transcription factor) [18]. Loss of KCNQ1OT1 inhibited BRCA cell proliferation and migration in BT-549 and HCC1599 cells and reduced tumor growth in vivo by sponging miR-107 [26] (Table 1). Additional experiments are needed to investigate whether cyclin-dependent kinase 8 (CDK8) is involved in the epigenetic regulation of the KCNQ1OT1–hsa-miR-107 axis, as CDK8 has been previously shown by Li et al. to regulate miR-107 in BRCA [76].

KCNQ1OT1 serves as ceRNA to regulate multidrug resistance via regulating miR-27b-3p/activating transcription factor 2 (ATF2) in human chordoma bone tumor cells [24]. Zhu et al. hypothesized that KCNQ1OT1 is correlated with poor prognosis in patients with soft tissue sarcoma (STS), competitively binds with miR-29c-3p, regulating JARID2, CDK6, DNMT3A, and TET [13] (Table 1). Thus, this intricate ceRNA network may serve as a therapeutic target for treating the STS sub-cluster of patients with a poor prognosis. KCNQ1OT1 upregulation induced cell proliferation and migration and inhibited apoptosis in HOS and U2OS OS cells through competitive binding of miR-4458 and upregulating cyclin D2 (CCND2) [21]. KCNQ1OT1 also promoted U2OS and 143B OS cell proliferation by enhancing aerobic glycolysis through competitive binding to miR-34c-5p and stimulating aldolase A (ALDOA) expression in vitro and in vivo [22] (Table 1).

KCNQ1OT1 acts as a competing endogenous RNA (ceRNA) for miR-145-5p, resulting in increased expression of poly(rC)-binding protein 2 (PCBP2). PCBP2 is a target of miR-145-5p, and its overexpression results in the progression of BC by modulating cell proliferation, migration and invasion, and cell apoptosis [32] (Table 1). Therefore, KCNQ1OT1 expression may identify the subset of BC patients with a more aggressive phenotype. Wang et al., have shown that KCNQ1OT1 also sponged miR-129-5p and regulated jagged canonical Notch ligand 1 (JAG1) expression that induces proliferation, migration, and invasion of A549 and H460 NSCLC cells [36] (Table 1). KCNQ1OT1 was upregulated in irradiation-resistant LAD cells and is associated with the low response to anticancer treatment and poor prognosis of LAD patients [37]. Knockdown in stereotactic body radiation therapy-resistant cells significantly enhanced radiosensitivity both in vitro and in vivo by sponging miR-372-3p and regulating autophagy-related targets (ATG5 and ATG12), thereby inhibiting autophagy (Table 1). KCNQ1OT1 is aberrantly upregulated in melanoma and retinoblastoma (RB) patient tissues compared with adjacent normal tissues [41,77,78]. Overexpression of KCNQ1OT1 contributed to the proliferation, migration, and invasion of melanoma and RB cells by sponging miR-153, and increasing MET proto-oncogene receptor tyrosine kinase (MET) and hypoxia-inducible factor-1α (HIF-1α) expression, respectively [41,77] (Table 1). KCNQ1OT1 also acts as a ceRNA for miR-124 to promote RB cell progression by regulating the transcription factor, specificity protein 1 (SP1) expression, and the silent information regulator 1 (SIRT1)/c-Jun N-terminal kinase (JNK) signaling pathway [78].

KCNQ1OT1 was highly expressed in CRC, and its knockdown suppressed cell proliferation, migration, and invasion by interacting with miR145-5p/zinc finger protein 146 (ZNF146) [5]. In addition, Wang et al. found that KCNQ1OT1 promoted GC progression by sponging miR-4319 to upregulate the expression of DNA-damage-regulated autophagy modulator 2 (DRAM2) [46]. Moreover, KCNQ1OT1 is discussed as a ceRNA for miR-148a-3p and a positive regulator for IGF1R in HCC [31].

In a recent study by Chen et al., KCNQ1OT1, PD-L1, and CD8 levels were significantly increased in prostate cancer tissues compared with adjacent non-tumor tissues [42]. KCNQ1OT1 was shown to regulate PD-L1 expression by sponging miR-15a in PCa, resulting in the inhibition of cytotoxicity of CD8+ T cells and promotion of tumor evasion (Table 1). Furthermore, knockdown of KCNQ1OT1 significantly decreased PD-L1 expression, inhibited the viability, migration, invasion, and EMT, promoted apoptosis of PCa cells, and enhanced the function of CD8+ T cells. Zhang et al., further demonstrated that knockdown of KCNQ1OT1 could reduce sorafenib resistance and PD-L1-mediated immune escape, regulate cytokine secretion and CD8+ T-cell apoptosis, and suppress migration and invasion in sorafenib-resistant HCC cells by sponging miR-506 [30]. These findings indicate that KCNQ1OT1 plays a significant oncogenic role in PCa and HCC tumorigenesis and may become a promising therapy that targets tumor evasion and drug resistance and inhibits the malignant growth of cells.

KCNQ1OT1 expression was positively associated with Chitinase 3 Like 1 (CHI3L1) expression and significantly promotes prostate cancer (PCa) cell proliferation, invasion, and metastasis [43]. Hao et al. found that overexpression of KCNQ1OT1 competes with miR-211-5p expression, which functions as a ceRNA to promote CHI3L1 expression and PCa progression [43] (Table 1). These results suggest that KCNQ1OT1 may be a prognostic marker for poor outcomes in PCa. In contrast, Li et al., reported that overexpression of KCNQ1OT1 promoted apoptosis in neuroblastoma cells by sponging miR296-5p and upregulating BCL2 Associated X (Bax), a key regulator of cell death [12], suggesting a cancer cell type-dependent role of KCNQ1OT1.

### 4.2. Impact of KCNQ1OT1 on Cell Signaling Pathways

An increasing number of studies have demonstrated that lncRNAs modulate oncogenic signaling [79,80], highlighting their utility as diagnostic markers and therapeutic targets [14]. Several studies have revealed that KCNQ1OT1 is overexpressed in SCLC patients and is associated with a poor prognosis. Downregulation of KCNQ1OT1 inhibits SCLC cell proliferation, migration, and invasion, induces apoptosis, and suppresses tumor growth and chemoresistance via TGF-β-mediated EMT signaling [73] and the Janus kinase (JAK)/signal transducer and activator of transcription 3 (STAT3) signaling pathway [72]. Duan et al. reported that KCNQ1OT1 promoted the malignancy SW620 and RKO CRC cells by upregulation of the PI3K/AKT signaling pathway [34]. Moreover, KCNQ1OT1 promoted OS cell proliferation, migration, invasion, and EMT in primary osteosarcoma cells by activating β-catenin [54].

## 5. Conclusions and Future Perspectives

LncRNAs have been shown to play essential roles in regulating gene expression and the development and progression of cancers. In the current review, we summarize what is currently known regarding the roles of KCNQ1OT1 in a wide range of human cancers. We report that KCNQ1OT1 functions as a signaling lncRNA by regulating gene expression in cell signaling pathways and as an oncogenic lncRNA through interacting with miRNAs as a ceRNA. Participation in the ceRNA regulatory network is a major mechanism of KCNQ1OT1 activity in tumor development and progression. KCNQ1OT1 competitively binds with numerous microRNAs, such as miR-129-5p, miR-145, miR-148a-3p, miR-211-5p, miR-217, miR-204, miR-326, miR-9, miR-506, miR-4458, miR-140-5p, miR-370, and miR-372-3p, resulting in the aberrant expression of their downstream target genes, regulating multiple signaling pathways in cancers as summarized in Table 1. Aberrant expression of KCNQ1OT1 is associated with various types of cancers. However, target genes vary and are cancer cell type-dependent (Figure 2).

**Figure 3:**
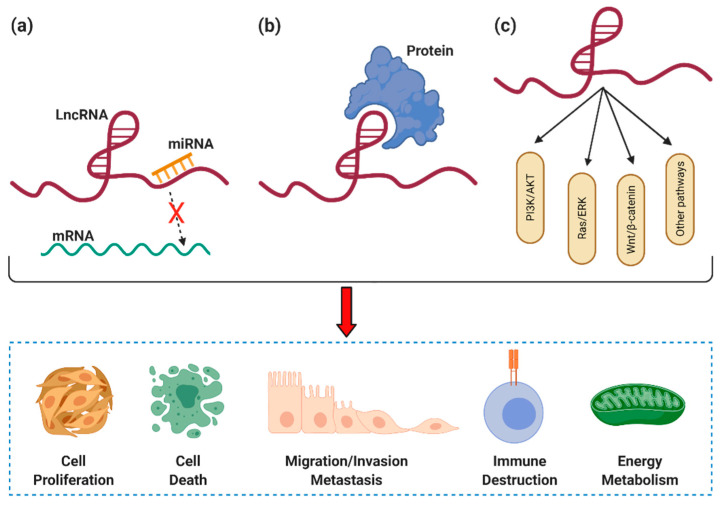
Mechanisms of KCNQ1OT1 in cancer. (**a**) KCNQ1OT1 interacts with miRNAs as ceRNAs, reducing the interaction between the miRNA and mRNA; (**b**) KCNQ1OT1 binds with target proteins, and (**c**) KCNQ1OT1 participates in the transduction of different signal pathways. Then, KCNQ1OT1 can exert its function to regulate tumorigenesis by influencing cancer hallmarks. Created with BioRender.com.

LncRNA KCNQ1OT1 acts as an oncogene and can alter cellular processes. The mechanisms by which KCNQ1OT1 promotes tumorigenesis are complex and involve multiple steps, including promoting proliferation/migration/invasion/EMT by competitively binding to miRNAs and activating PI3K/AKT, Ras/ERK, Wnt/β-catenin, apoptosis/autophagy, and hippo signaling pathways (Figure 3). Interestingly, KCNQ1OT1 has been identified as a suppressor of gastric cancer cell proliferation, migration, and invasion. Overexpression of KCNQ1OT1 inhibits cell proliferation in the early stages of NSCLC and is correlated with better prognosis in NSCLC patients, suggesting a unique link with patient prognosis.

The diversity of roles of lncRNA KCNQ1OT1 in cancer is likely due to factors such as tumor tissue origin and stage, extracellular microenvironment, and the presence of other regulatory molecules. Recent studies show that lncRNA KCNQ1OT1 can function not only as an oncogene but as a tumor suppressor. KCNQ1OT1 also promotes other malignant behaviors, including regulating key enzymes in metabolic pathways, stimulating aerobic glycolysis, and promoting tumor evasion by inhibiting the function of CD8+ T cells. It will be informative to use KCNQ1OT1 knockout mice or an orthotopic mouse model of cancer invasion and metastasis using KCNQ1OT1-depleted cultured cell lines to identify conditions under which the functions of KCNQ1OT1 are critical. Human-patient-derived xenograft model studies that investigate whether targeting KCNQ1OT1 impairs cancer development could also potentially shed more light on the role of KCNQ1OT1 as a therapeutic target.

Identification of molecular biomarkers is essential for diagnosis and prognosis determination in cancer patients. Although several studies have detected lncRNAs in circulation, the expression level and chemical stability of KCNQ1OT1 have not been evaluated in other body fluids. Therefore, if KCNQ1OT1 could be detected in the urine or serum of cancer patients, it could be developed as a biomarker for early detection and late-stage cancer patients.

In conclusion, current studies indicate that the dysregulation of KCNQ1OT1 is a significant factor to consider in the diagnosis, prognosis, treatment, and response to therapy for patients with a wide range of cancer types.

## Figures and Tables

**Table 1:** Functional characterization of lncRNA KCNQ1OT1 and its targets in various cancers.

Cancer	Expression Level	Role	Associated Clinical Features †	Functional Role †	Regulatory Molecule and Pathway [Reference]
Colorectal cancer	Upregulated	Oncogenic	Tumor size, TNM stage, lymph node metastasis, distant metastasis, histological differentiation, adjuvant therapy, primary tumor site, OS, DFS	Proliferation, cell cycle, apoptosis, migration, invasion, aerobic glycolysis, methotrexate resistance, adjuvant fluoropyrimidine-based chemotherapy	miR-217/ZEB1 [1]; miR-34a/Atg4B [2]; miR-760/PPP1R1B [3]; miR-329-3p/CTNND1 [4]; miR-216b-5p/ZNF146 [5]; HK2 [6]; PI3K/AKT [7]; CD155 [8]
Ovarian cancer	Upregulated	Oncogenic	OS	Proliferation, invasion,	miR-142-5p/CAPN10 [9]
Cervical cancer	Upregulated	Oncogenic	not investigated	Proliferation, metastasis, and radioresistance	miR-491-5p/PKM2 [10]
Glioma	Upregulated	Oncogenic	Histopathological grade	Proliferation, apoptosis, migration, invasion	miR-370/CCNE2 [11]
Neuroblastoma	not investigated	Suppressor	not investigated	Apoptosis	miR-296-5p/Bax [12]
Sarcoma	not investigated	not investigated	Histological type, metastasis, tumor depth, necrosis	not investigated	miR-29c-3p)/JARID2/CDK8/DNMT3A/TET1 [13]
Oral squamous cell carcinoma	Upregulated	Oncogenic	not investigated	Apoptosis, migration, invasion	miR-185-5p/Rab14 [14]
Tongue squamous cell carcinoma	Upregulated	Oncogenic	Clinical stage, node metastasis, survival status, cisplastin sensitivity	Proliferation, migration, invasion, cisplatin resistance	miR-211-5p/Ezrin/Fak/Src [15]; miR-124-3p/TRIM14 [16]
Maxillary sinus squamous cell carcinoma	Upregulated	Oncogenic	not investigated	Viability, migration, invasion,	miR-204/EphA7 [17]
Acute myeloid leukemia	Upregulated	Oncogenic	not investigated	Proliferation, apoptosis, PMA-induced differentiation	miR-326/c-Myc [18]
Osteosarcoma	Upregulated	Oncogenic	not investigated	Proliferation, apoptosis, migration, invasion, EMT, aerobic gylcosis, fluorouracil resistance,	β-catenin [19]; KCNQ1/DNMT1 [20]; miR-4458/CCND2 [21]; miR-34c-5p/ALDOA [22]; miR-192-5p/LARP1 [23]
Chordoma	Upregulated	Oncogenic	not investigated	Multidrug resistance	miR-27b-3p/ATF2 [24]
Breast cancer	Upregulated	Oncogenic	Tumor size, tumor count, tumor stage	Proliferation, cell cycle, apoptosis, migration	miR-145/CCNE2 [25]; miR-107 [26]
Cholangiocarcinoma	Upregulated	Oncogenic	Tumor site, differentiation grade, tumor stage, TMN stage, lymph node metastasis, postoperative recurrence	Proliferation, apoptosis, invasion, EMT	miR-140-5p/SOX4 [27]
Hepatocellular carcinoma	Upregulated	Oncogenic	not investigated	Proliferation, viability, survival, apoptosis, migration, invasion, metastasis, oxaliplatin and sorafenib resistance	miR-7-5p/ABCC1 [28]; miR-149/S1PR1/PI3K/AKT [29]; miR-506/PD-L1 [30]; miR-148a-3p/IGF1R [31]
Bladder cancer	Upregulated	Oncogenic	Poor prognosis	Proliferation, apoptosis, migration, invasion	miR-145-5p/PCBP2 [32]
Lung cancer	Upregulated	Oncogenic	Tumor size, TNM stage, disease stage, lymph node metastasis, histological differentiation, smoking history, OS	Proliferation, cell cycle, autophagy, apoptosis, migration, invasion, aerobic glycolysis, multidrug resistance, irradiation resistance	miR-204-5p/ATG3 [33]; MDR1 [34]; miR-27b-3p/HSP90AA1 [35]; miR-129-5p/JAG1 [36]; miR-372-3p/ATG5/ATG12 [37]; JAK2/STAT3 [38]; TGF-β1 [39]
Lung cancer	Upregulated	Suppressor	Clinical stage, tumor size, lymph node metastasis	Proliferation	not investigated [40]
Melanoma	Upregulated	Oncogenic	Poor prognosis	Proliferation, metastasis	miR-153/MET [41]
Prostate cancer	Upregulated	Oncogenic	not investigated	Proliferation, apoptosis, migration, invasion, metastasis	miR-15a/PD-L1 [42]; miR-211-5p/CHI3L1 [43]
Gastric cancer	Upregulated/Downregulated	Oncogenic/Suppressor	TNM stage, local invasion, lymph node metastasis, distant metastasis, histological grade	Proliferation, viability, survival, apoptosis, migration, invasion	miR-9/LMX1A [44]; miR-145-5p/ARF6 [45]; miR-43198/DRAM2 [46]

Key †: TNM (tumor lymph node metastasis), overall survival (OS), disease-free survival (DFS), epithelial–mesenchymal transition (EMT).

## Data Availability

Not applicable.
